# A Case of Non-Small Cell Lung Cancer with Mutually Exclusive *EGFR* and *KRAS* Mutations

**DOI:** 10.3390/cimb47010066

**Published:** 2025-01-20

**Authors:** Abhimanyu Tushir, Israh Akhtar, Anjali Seth

**Affiliations:** Department of Pathology and Laboratory Medicine, Temple University Hospital, Philadelphia, PA 19140, USA; abhimanyu.tushir@tuhs.temple.edu (A.T.); israh.akhtar@tuhs.temple.edu (I.A.)

**Keywords:** next-generation sequencing, *KRAS*, *EGFR*, non-small cell carcinoma

## Abstract

Historically, *EGFR* and *KRAS* mutations were believed to be mutually exclusive. However, over the past few years, there have been emerging case reports showing the co-existence of both mutations in a single case. The majority of these co-occurring alterations were detected in samples collected from patients with resistance to tyrosine kinase inhibitor (TKI) treatment, indicating a potential functional role in driving resistance to therapy. These co-occurring tumor genomic alterations are not necessarily mutually exclusive, and evidence suggests that multiple clonal and sub-clonal cancer cell populations can co-exist and contribute to *EGFR* TKI resistance. We have reported such a case of concomitant *EGFR* and *KRAS* mutation in a 64-year-old female. This case highlights the importance of continuous molecular testing in managing NSCLC, especially in cases with rare mutation profiles. The emergence of new mutations during treatment can significantly impact the course of therapy and patient outcomes. In this case, the detection of both *EGFR* and *KRAS* mutations guided the selection of an appropriate targeted therapeutic strategy, including the use of Amivantamab.

## 1. Introduction

Non-small cell lung cancer (NSCLC) accounts for 80–85% of all lung cancer cases and is a leading cause of cancer-related deaths worldwide [1,2]. It primarily affects older adults, with smoking being a significant risk factor. However, non-smokers with driver gene mutations, such as *EGFR* are often diagnosed at advanced stages, which contributes to lower survival rates.

Molecular profiling has transformed NSCLC diagnosis and treatment. The discovery of genetic mutations, especially through next-generation sequencing (NGS), has enabled targeted therapies, improving outcomes for patients [3]. The most common driver mutations are *EGFR* (present in 45% of Asian and 20% of Caucasian adenocarcinoma patients) and *KRAS* (found in 25% of adenocarcinomas) [3,4]. *EGFR* mutations are more common in non-smokers and respond well to tyrosine kinase inhibitors (TKIs), while *KRAS* mutations, more common in smokers, are resistant to TKI therapy and associated with poorer outcomes [5,6].

While *EGFR* and *KRAS* mutations were once thought to be mutually exclusive, recent reports have shown cases where both mutations coexist, particularly in patients who develop resistance to TKIs [7,8]. These co-occurring mutations may drive resistance, but studies indicate no survival benefit from their coexistence. Despite the emergence of *KRAS* mutations in *EGFR* (+) NSCLCs at disease progression, their role in the response to *EGFR* tyrosine kinase inhibitors (TKIs) remains marginally explored [9]. Our case report contributes to the existing literature on coexisting *KRAS* and *EGFR* mutations and their impact on clinical outcomes.

## 2. Case Report

A 64-year-old woman with a past medical history of chronic renal disease, headaches, hypertension, and depression presented to the urgent care clinic in December 2020 with complaints of pain in her left ribs and back that worsened during breathing. On examination, this area was tender. There was no history of injury, shortness of breath, or weight loss. She was a past smoker with no family history of cancer.

A lidocaine patch was prescribed, and a chest X-ray was ordered, which showed a “mass like” density in the left lung base. A chest computer–tomography (CT-scan) was ordered which revealed a 4.4 × 4.6 × 3.3 cm mass in the left lower lobe, highly suspicious for malignancy. In addition, numerous bilateral pulmonary nodules and mildly enlarged mediastinal lymph nodes, suspicious for metastatic disease, were noted. A PET scan confirmed several prominent mediastinal and subcarinal lymph nodes and metabolically active lytic lesions in multiple areas of the spine.

An endobronchial ultrasound-guided fine needle aspiration of the station 11L (interlobar/lobar) lymph node and station 10L (hilar lymph node) were performed. Station 11L was positive for metastatic non-small cell carcinoma. However, the tumor cells in the cell block were insufficient for ancillary and molecular testing. Station 10L had only a few atypical cells and was suboptimal for definitive diagnosis or ancillary testing.

A CT-guided core biopsy of the lung mass was then ordered, which confirmed the diagnosis of pulmonary adenocarcinoma. PDL-1 testing was positive with a >1% TPS (tumor proportion score) score.

Molecular testing was inadequate due to limited tissue. However, a liquid biopsy was performed which revealed an *EGFR* L858R mutation at a variant allele frequency of 0.44%. The *EGFR* L858R mutation is a missense alteration located within the kinase domain of the EGFR protein. It is one of the most common mutations in non-small cell lung cancer (NSCLC) and predicts sensitivity to *EGFR*-targeted therapies.

In January 2021, the patient received a combination of pembrolizumab, pemetrexed, and carboplatin chemotherapy. After four cycles, imaging revealed a significant reduction in the size of the lung mass. Osimertinib was started, which led to further reduction in the tumor size.

The patient was compliant and received her treatment as scheduled. She tolerated her chemotherapy well, except for mild constipation. However, an MRI of the brain was performed in September 2022, which showed two brain lesions: one in the superior right frontal lobe and the other in the right parietal lobe, as well as lesions in the calvarial bone consistent with metastases. The chemotherapy was continued, and a gamma knife surgery was initiated for intracranial metastases.

**Figure 1:**
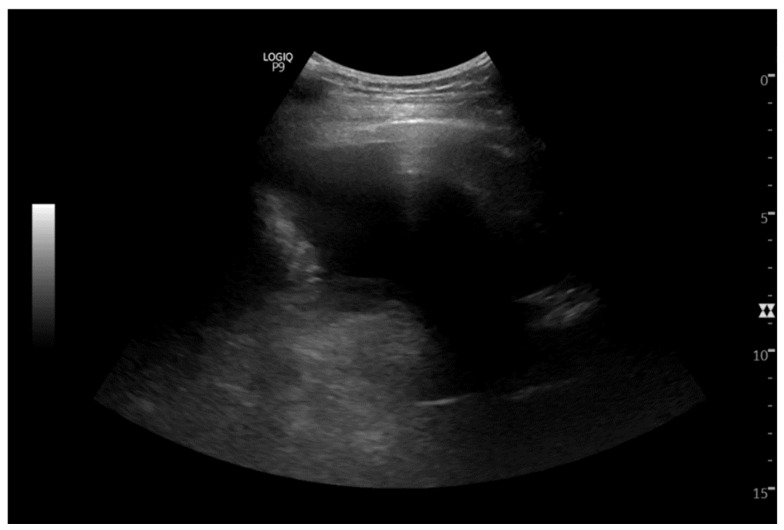
Ultrasound demonstrating a moderate left pleural effusion. Ultrasound-guided thoracocentesis was performed.

The patient with T4N2M1c stage IVB lung cancer was responding well to chemotherapy and showed a decrease in the left lower lobe mass, now measuring 2.2 × 1.9 cm, and her CT and MRI showed no evidence of progression of the disease. However, by March 2023, she complained of shortness of breath and a repeat CT-scan showed a small pericardial effusion and multilobulated 4.8 × 4.2 cm left lower lobe perihilar soft tissue mass lesion (Figure 1). The dose of Osimertinib was increased to 80 mg daily, which was well-tolerated by the patient.

**Figure 2:**
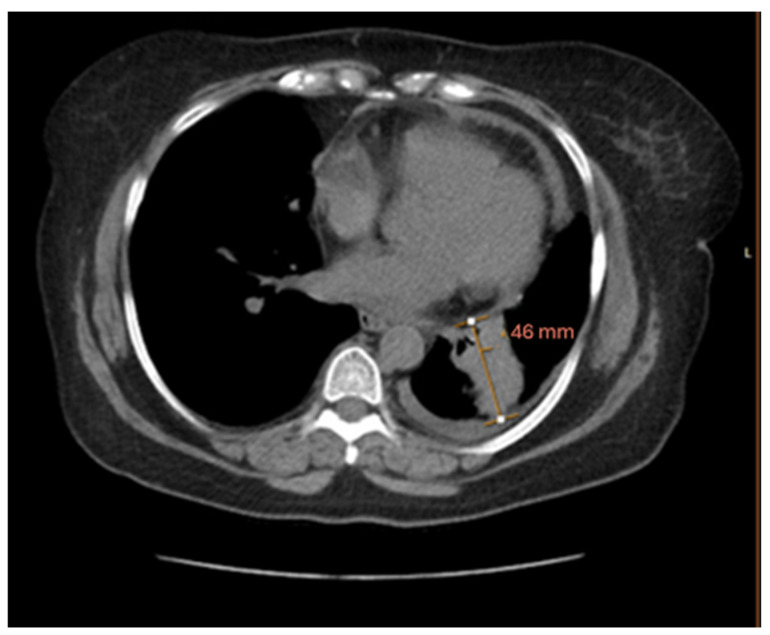
CT-scan at the time of diagnosis showing a left lower lobe perihilar soft tissue mass lesion.

**Figure 3:**
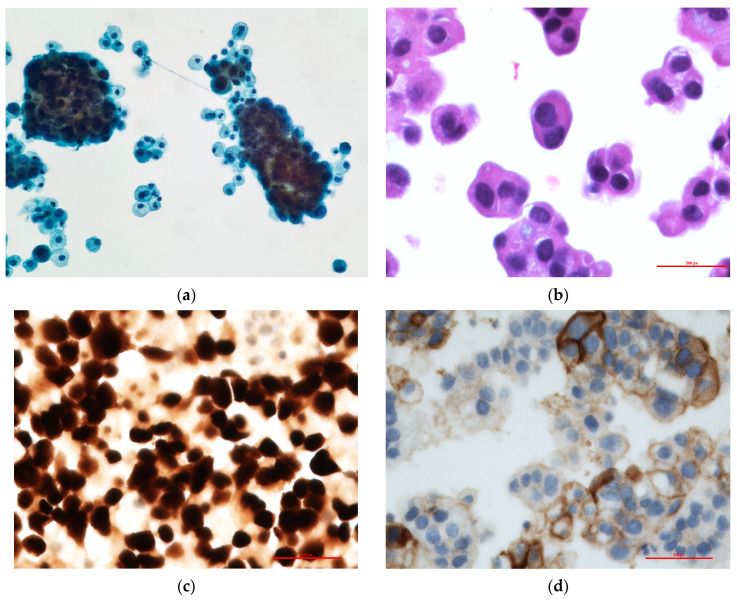
Pleural fluid sample. (**a**) Papanicolaou stain 200×, Thin Prep. shows cellular specimen with numerous atypical clusters of cells. (**b**) Cell block (Hematoxylin and eosin stain) 500×. shows clusters of tumor cells consistent with metastatic adenocarcinoma. (**c**) TTF-1 stain 400×. Tumor cells stained with TTF-1 confirming metastatic lung adenocarcinoma. (**d**) PDL-1 immunostain 400×. Membrane staining of a portion of tumor cells in cell block.

By May 2024, the patient developed moderate pleural effusion and 400 cc of pale-yellow fluid was obtained by thoracentesis that was submitted for cytological analysis. (Figure 2). The cytological examination revealed metastatic adenocarcinoma (Figure 3a,b). The cell block contained adequate tumor cells for ancillary testing and next-generation sequencing. The TTF-1 clone 8G7G3/1 was positive, with strong nuclear staining, confirming metastasis from the lung primary (Figure 3c). The PDL-1 clone 22C3 tumor proportion score was 40% (Figure 3d).

Next-generation sequencing (NGS) revealed both *EGFR* L858R and *KRAS* G12D mutations in June 2024. For DNA NGS, tumor DNA was used for multiplex amplification with gene-specific primers targeting the coding exons and exon flanking regions of 275 cancer-related genes using the QIAseq Human Comprehensive Cancer Panel (Qiagen, Inc., Hilden, Germany) NGS was performed using the NextSeq2000 (Illumina, San Diego, CA, USA) and analyzed with the CLC Genomics Workbench (Qiagen). The variants were filtered based on variant allele frequency (VAF, ≥5%), mutation databases, and population-based reference datasets including gnoAD, ClinVar, and COSMIC. NGS confirmed this mutational change, with *EGFR* L858R having a VAF of 49.50%, and also detected the presence of *KRAS* G12D at a VAF of 49.27%. The *KRAS* G12D mutation is an activating alteration within the *KRAS* gene, a gene commonly mutated in several malignancies including lung adenocarcinoma. *KRAS* mutations are associated with poor prognosis in NSCLC and are generally mutually exclusive with *EGFR* mutations. *KRAS* G12D has been reported in approximately 20% of lung adenocarcinoma cases. Three variants of unknown significance were also detected in the patient specimen. These were *ALK* R1436H (VAF 23.2%), *LRP1B* N2814D (40.2%), and *KMT2D* S2251L (23.2%). *ALK* R1436H has not been functionally characterized and its effect on protein function is unknown. Although *LRP1B* N2814D and *KMT2D* S2251L have not been functionally characterized, mutations in these two genes have been reported in lung adenocarcinomas.

Targeted NGS RNA fusion analysis using a custom Illumina TruSight 527 gene next-generation sequencing panel did not reveal RNA fusions.

Following these findings, standard of care treatment options were discussed, a change in therapy was made, and the patient was started on Amivantamab. The patient was offered enrollment in clinical trials but declined the offer.

Despite the change in therapy, the patient again presented to the hospital within two months of treatment with nausea, vomiting, and bloody diarrhea. She was admitted for pancytopenia and failure to thrive in September 2024. The symptoms were suspected to be related to recent chemotherapy. The clinical condition continued to deteriorate despite all efforts, and the patient passed away while on comfort care.

## 3. Discussion

Next-generation sequencing (NGS) has significantly advanced the detection of concomitant driver alterations in non-small cell lung cancer (NSCLC) [9,10], uncovering the presence of clonal and sub-clonal populations within tumors that contribute to resistance against tyrosine kinase inhibitor (TKI) therapies. Among these, the coexistence of *EGFR* and *KRAS* mutations poses a substantial therapeutic challenge. While *KRAS* mutations are well-documented drivers of primary resistance to EGFR-TKIs in NSCLC, their role in acquired resistance among *EGFR*-mutant patients is less well-defined.

Interestingly, studies have shown that some patients with both *EGFR* and *KRAS* mutations respond to EGFR-TKI therapy, such as Icotinib, but not to other targeted therapies like crizotinib. This suggests that *EGFR* may remain the dominant oncogenic driver in certain cases, even in the presence of *KRAS* mutations. However, tumors are often heterogeneous, with *EGFR*-mutated cells responding to treatment while *KRAS*-mutant cells drive resistance, resulting in mixed clinical outcomes.

The prevalence of coexisting *KRAS* and *EGFR* mutations appears to increase following *EGFR*-TKI therapy. For instance, one study reported that the prevalence of dual mutations rose to 3.49% after TKI treatment [11], supporting the hypothesis that dormant tumor cells harboring both mutations may proliferate after *EGFR* inhibition. Patients with *KRAS* mutations, particularly at codon 12 (e.g., G12D, G12V, and G12C), exhibit poor responses to treatment, highlighting the aggressive nature of these alterations [12,13].

Several clinical studies have investigated the impact of *KRAS* mutations on treatment outcomes. Benesova et al. documented limited progression-free survival (PFS) in patients with *EGFR/KRAS* co-mutations, ranging from 3 to 7 months [14]. Similarly, Ranchiglio et al. reported significantly shorter PFS (2.42 months) and lower objective response rates (ORR) in patients with dominant *KRAS* mutations compared to those with *EGFR* mutations alone [15]. Nardo et al. observed poor clinical outcomes in patients with low variant allele frequencies (VAF < 0.2%) of KRAS mutations, with treatment failure times and PFS of approximately 5 months [16].

Other studies have confirmed the complex relationship between *EGFR* and *KRAS* co-mutations. For example, Zhuang et al. analyzed 3774 patients and found that *EGFR*/*KRAS* co-mutants had an ORR of 62.5% to *EGFR*-TKI therapy, with no statistically significant difference in PFS compared to patients with single *EGFR* mutations. However, these findings were inconsistent across studies, with some reporting worse outcomes in co-mutated cases [17].

In cases where *KRAS* hyper-exchange mutations disrupt the regulatory function of GTPase-activating proteins (GAPs), *KRAS* remains in a continuously active state, fueling uncontrolled tumor growth and promoting resistance [18]. Dual inhibition strategies targeting both *EGFR* and downstream pathways, such as the MEK pathway, have been proposed to overcome resistance in *EGFR* mutant tumors with *KRAS* mutations.

In the case of our patient, the *KRAS* G12D mutation likely emerged after prolonged TKI therapy. By mid-2024, the presence of dual *EGFR* and *KRAS* mutations led to a shift in treatment to Amivantamab, a dual inhibitor targeting *EGFR* and MEK pathways. While this provided temporary stabilization, the aggressive nature of the *KRAS* G12D mutation ultimately contributed to treatment failure and poor outcomes.

This case underscores the complexity of managing NSCLC with coexisting *EGFR* and *KRAS* mutations and highlights the importance of continuous molecular monitoring to adapt treatment strategies as the cancer evolves.

## 4. Conclusions

This case highlights the importance of continuous molecular testing in managing NSCLC, especially in cases with rare mutation profiles. The emergence of new mutations during treatment can significantly impact the course of therapy and patient outcomes. In this case, the detection of both *EGFR* and *KRAS* mutations guided the selection of a more personalized targeted therapeutic strategy, including the use of Amivantamab. By utilizing Amivantamab, a bispecific antibody targeting *EGFR* and MET, the therapy was tailored to address the complexity of the patient’s molecular profile. This highlights the potential for targeted treatments to provide better disease control and improved outcomes in NSCLC patients with co-occurring mutations.

Additionally, this case reinforces the need for clinicians to remain vigilant through regular molecular monitoring, ensuring that therapeutic strategies are continually optimized as the genetic landscape of the tumor evolves over time.

## Data Availability

Data are contained within the article.
